# Protection of Mice from Acute Graft-versus-Host Disease Requires CD28 Co-stimulation on Donor CD4^+^ Foxp3^+^ Regulatory T Cells

**DOI:** 10.3389/fimmu.2017.00721

**Published:** 2017-06-23

**Authors:** Anna Uri, Sandra Werner, Fred Lühder, Thomas Hünig, Thomas Kerkau, Niklas Beyersdorf

**Affiliations:** ^1^Institute for Virology and Immunobiology, University of Würzburg, Würzburg, Germany; ^2^Institute for Multiple Sclerosis Research and Neuroimmunology, University Medical Centre Göttingen, Göttingen, Germany

**Keywords:** acute graft-versus-host disease, CD28, co-stimulation, regulatory T cells, inducible deletion

## Abstract

Acute graft-versus-host disease (aGvHD) is a major cause of morbidity and mortality after allogeneic hematopoietic stem cell plus T cell transplantation (allo-HSCT). In this study, we investigated the requirement for CD28 co-stimulation of donor CD4^+^ conventional (CD4^+^CD25^−^Foxp3^−^, Tconv) and regulatory (CD4^+^CD25^+^Foxp3^+^, Treg) T cells in aGvHD using tamoxifen-inducible CD28 knockout (iCD28KO) or wild-type (wt) littermates as donors of CD4^+^ Tconv and Treg. In the highly inflammatory C57BL/6 into BALB/c allo-HSCT transplantation model, CD28 depletion on donor CD4^+^ Tconv reduced clinical signs of aGvHD, but did not significantly prolong survival of the recipient mice. Selective depletion of CD28 on donor Treg did not abrogate protection of recipient mice from aGvHD until about day 20 after allo-HSCT. Later, however, the pool of CD28-depleted Treg drastically declined as compared to wt Treg. Consequently, only wt, but not CD28-deficient, Treg were able to continuously suppress aGvHD and induce long-term survival of the recipient mice. To our knowledge, this is the first study that specifically evaluates the impact of CD28 expression on donor Treg in aGvHD. Moreover, the delayed kinetics of aGvHD lethality after transplantation of iCD28KO Treg provides a novel animal model for similar disease courses found in patients after allo-HSCT.

## Introduction

Inherited disorders or neoplastic malignancies of the hematopoietic system can be efficiently cured by allogeneic hematopoietic stem cell plus T cell transplantation (allo-HSCT). Mature donor T cells contained in the graft enhance engraftment of hematopoietic stem cells, protect from opportunistic infections, and also mediate the beneficial graft-versus-leukemia (GvL) effect (1). However, donor T cells also cause acute graft-versus-host disease (aGvHD) (2). General immunosuppression is the standard treatment for aGvHD, but it also dampens the GvL response (1). A promising strategy to overcome this dilemma is based on interfering with the function of costimulatory molecules like CD28 on donor T cells (3). CD28 co-stimulation (“signal 2”) (4) regulates the balance between T cell activation and tolerance in various ways: anergy induction in the absence of CD28 co-stimulation is one mechanism to mediate tolerance of T cells against self-peptides (5). However, a highly inflammatory environment or strong T cell receptor signaling can overcome this need for CD28 co-stimulation for effective T cell activation (6–10). In this context, the so-called “signal 3” originating from other costimulatory molecules like Ox40 and 4-1BB or receptors for cytokines like IL-2, TNF, IL-12, and IL-6 might, at least partially, compensate for a lack in CD28 signaling (11). In addition, thymic natural (12) and peripherally induced (13) CD4^+^ CD25^+^ Foxp3^+^ regulatory T cell (Treg) development as well as peripheral homeostasis of Treg under steady-state conditions requires CD28 co-stimulation (14–17). Treg have, further, been shown to suppress aGvHD (18–23) without abrogating the GvT effect (18–24).

Thus, even though the requirement for CD28 co-stimulation in sustaining immune homeostasis in a steady-state situation is well known, the role of co-stimulation by CD28 for Tconv and especially Treg function in an inflammatory environment still remains unclear. We used the C57BL/6 into BALB/c aGvHD model to study T cell co-stimulation under highly inflammatory conditions, i.e., in the first week after transplantation, and under steady state-like conditions, i.e., during the late phase of the disease, when inflammatory damage due to the conditioning regime has mostly subsided and the host has become allotolerant (2, 25, 26). We isolated T cells from tamoxifen-inducible CD28 knockout (iCD28KO) mice in this aGvHD model in a way that CD28 could be inducibly deleted on different donor T cell subsets. Using T cells from iCD28KO mice avoids the problem of altered thymic differentiation observed in conventional CD28^−/−^ mice (12, 27–29). Moreover, iCD28KO mice are also superior to approaches using anti-CD28 antibodies to interfere with CD28 function due to agonistic effects of intact antibodies and low affinity of Fab fragments (30, 31). With our novel approach outlined here, we addressed the following questions: (1) Can Tconv induce aGvHD after CD28 deletion? (2) Is CD28 co-stimulation of Treg required for suppression of Tconv and long-term survival of recipient mice?

## Materials and Methods

### Animals

Inducible CD28 knockout mice (B6.Thy1.1^+/−^ ErCre^+/−^ CD28^flox/−^) and their wt littermates (B6.Thy1.1^+/−^ ErCre^+/−^ CD28^+/−^) were generated by crossing B6.ERCre CD28^−/−^ mice that express the estrogen receptor fused Cre recombinase under the control of the Gt(ROSA)26Sor gene with mice that carry one floxed CD28 allele (B6.Thy1.1^+/+^ CD28 ^flox/+^). Thy1.1^+/+^ C57BL/6 mice were bred in the animal facility of the Institute for Virology and Immunobiology, University of Würzburg. Mice were used as T cell donors between 7 and 19 weeks of age. 8- to 9-week-old BALB/c OlaHsd recipient mice and C57BL/6J OlaHsd bone marrow donors were obtained from Envigo RMS GmbH (Venray, Netherlands).

### aGvHD Experiments

BALB/c recipient mice were given Neomycin (250 mg/l, Bela-pharm GmbH & Co. KG) and Polymyxin B (0.5 mg/l, Sigma-Aldrich) in their drinking water, beginning 4 days before until 27 days after transplantation in order to reduce the gut flora. BALB/c mice were lethally irradiated with a single dose of 8 Gy generated by a Faxitron X-ray source 24 h before intravenous transfer of 1 × 10^7^ T cell-depleted bone marrow (TCD-BM) cells from wild-type C57BL/6 mice and CD4^+^ T cells from either B6.Thy1.1^+^ mice, iCD28KO mice, or their wt littermates. TCD-BM wells were obtained by flushing femora and tibiae with BSS/0.1% BSA and, after erythrocyte lysis with TAC buffer (20 mM Tris, 155 mM NH_4_Cl, pH 7.2) and blockade of Fc receptors with 20 µg per ml of normal mouse Ig (Sigma-Aldrich), depleting CD90.2 positive T cells using magnetic activated cell sorting beads (Miltenyi Biotec, Bergisch Gladbach, Germany). CD4^+^ T cells were purified from peripheral and mesenteric lymph node (mLN) cells by negative selection (Miltenyi Biotec, or Affymetrix, Santa Clara, CA, USA). To obtain conventional CD4^+^CD25^−^ T cells, biotinylated anti-CD25 (clone 7D4, BD) antibody was directly added to the other biotinylated antibodies in the CD4^+^ negative selection kit (Miltenyi Biotec, Affymetrix). In order to obtain CD4^+^CD25^+^ Tregs, CD4^+^ T cells were stained with anti-CD25 (clone PC61) PE-conjugated antibody and positively selected with magnetic anti-PE beads (Miltenyi Biotec). In some experiments, donor T cells were labeled with 5 µM CFSE for 5 min at room temperature (RT) before transplantation. In order to deplete CD28 expression on transferred iCD28KO T cells with tamoxifen, a 40 mg pill (Hexal AG) was resolved in 3.2 ml drinking water and 100 µl of this solution were fed to the recipient mice by oral gavage on four consecutive days, complying to a daily dose of 1.25 mg tamoxifen, starting with the day of T cell transfer. In some experiments, CD28 was deleted in the donor mice by feeding them from day −4 to day −1 before transplantation with 100 µl of the same tamoxifen preparation. The clinical appearance of the recipient mice was scored by a blinded observer every other day as described previously (32). For each mouse, a cumulative score was calculated and mice with a cumulative score of eight or more were killed for humane reasons. Independent of the other clinical parameters, mice with less than 70% of their original body weight for more than 2 days and mice with a score of 2 in the category “spontaneous activity“ were killed.

### Fluorescence-Activated Cell Sorter Analysis (FACS)

The following antibodies and dyes were used for FACS analysis: anti-CD4 PE, anti-CD4 Pacific Blue, anti-CD4 Brilliant Violet 421, anti-CD90.1 Brilliant Violet 510, anti-Thy1.2 AlexaFluor 700, anti-CD28 (E18) APC, APC-conjugated mouse IgG_2b_ isotype control, anti-CD25 PE-Cy7 (PC61), anti-CD98 PE (all Biolegend), PE conjugated rat IgG_2a_, anti-CD25 FITC (PC61), anti-CD25 FITC (7D4), anti-Ki-67 PE (all BD Bioscience), anti-pAkt (Ser473) AlexaFluor 647 (Cell Signaling), anti-Foxp3 Percp-Cy5.5, viability dye eFluor780 (all ebioscience), anti-Foxp3 APC (Miltenyi Biotec), unconjugated glucose transporter 1 (Glut1) (ERP3915) (Abcam), and FITC-conjugated anti-rabbit IgG (Santa Cruz Biotechnology).

Stainings were performed with up to 10^6^ cells from mLNs, erythrocyte depleted spleen cells, or liver derived mononuclear cells, purified by Ficoll gradient, in 50 µl of FACS buffer [phosphate-buffered saline (PBS)/0.1% bovine serum albumin/0.02% NaN_3_]. Unspecific binding of flurochrome-conjugated antibodies was prevented by blocking FcγII/III receptors with supernatant of the clone 2.4G2 directed against CD16 and CD32. After surface staining (15 min, 4°C), cells were fixed for 30 min at RT (fixation buffer, eBioscience), permeabilized (permeabilization buffer, eBioscience), and intracellularly stained for Foxp3 and Ki-67 expression at RT for 45 min. For intracellular staining of Glut1 (33), cells were fixed and permeabilized, incubated with primary anti-Glut1 antibody (1:1,000, 45 min, RT), washed three times, incubated with FITC conjugated anti-rabbit IgG (45 min, RT) and blocked with normal mouse Ig (1:50) and normal rat serum (1:500) (15 min, RT) before staining for Foxp3 as described above. The cells were analyzed on a BD™ LSR II flow cytometer with the use of FACS Diva software (all Becton Dickinson). For further analyses of the data, FlowJo (TreeStar Inc.) software was used.

### Tracking of Alloreactive T Cell Expansion

In order to calculate absolute donor T cell numbers in spleens and mLNs and livers of recipient mice, total cells per organ were counted using trypan blue exclusion and multiplied with the percentages of donor CD4^+^ T cells as determined by FACS analysis.

### Analysis of Serum Cytokines

TNF concentrations were analyzed using the LEGENDPLEX bead-based immunoassay (Biolegend) according to the manufacturer’s instructions.

### Histology

Small and large bowels were fixed in 3.7% formalin, embedded in paraffin, and sections of 4 µm were stained with hematoxylin and eosin. Histopathological changes of the small bowel (lamina propria lymphocytic infiltrate, villous blunting, luminal sloughing of cellular debris, outright crypt destruction) and large bowel (lamina propria lymphocytic infiltrate, mucosal ulceration, outright crypt destruction) were graded by an observer blinded to the prior treatment as follows [scores in brackets; adapted from Hill et al. (34) and Cooke et al. (35)]: normal (0), focal and rare (0.5), focal and mild (1), diffuse and mild (2), diffuse and moderate (3), diffuse and severe (4). Scores for large and small bowel were cumulated into a single value.

For immunohistochemical staining, 4 µm paraffin sections were deparaffinized, boiled for 30 min in a citrate buffer (1.8 mM Citric Acid, 8.2 mM sodium citrate, pH 6.0) for antigen retrieval, blocked with 10% BSA/PBS and stained with anti-Foxp3 eFluor660 antibody (FJK-16s, ebioscience) in 1% BSA/PBS overnight. Slides were mounted in Roti-Mount Fluor Care (Roth) containing DAPI and fluorescence microscopy performed on a Leica DMi8 microscope equipped with an HCXPL FLUORTAR L 40×/0.60 DRY objective and a DFC3000G camera. Image acquisition and processing was performed using the LAS X software and the Image J software, respectively. Foxp3 and DAPI staining were detected in the LED-405 channel and Y5 channel, respectively. The RHOD Chanel was used to exclude autofluorescent signals from analysis. For quantitative analysis, the numbers of Foxp3 eFluor660 and DAPI double-positive cells in 10 high power fields of small and large bowel each were counted.

### Statistics

Summary graphs and statistical testing was done with GraphPad Prism 6.0d. *p* values of less than 0.05 were considered as statistically significant (*p < 0.05, **p < 0.01, ***p < 0.001).

### Study Approval

All experiments were performed in agreement with German law and approved by the Regierung von Unterfranken as the responsible authority.

## Results

### Donor Tconv Proliferation and Accumulation in the Host during aGvHD Is Normal Despite CD28 Deletion

**Figure 1:**
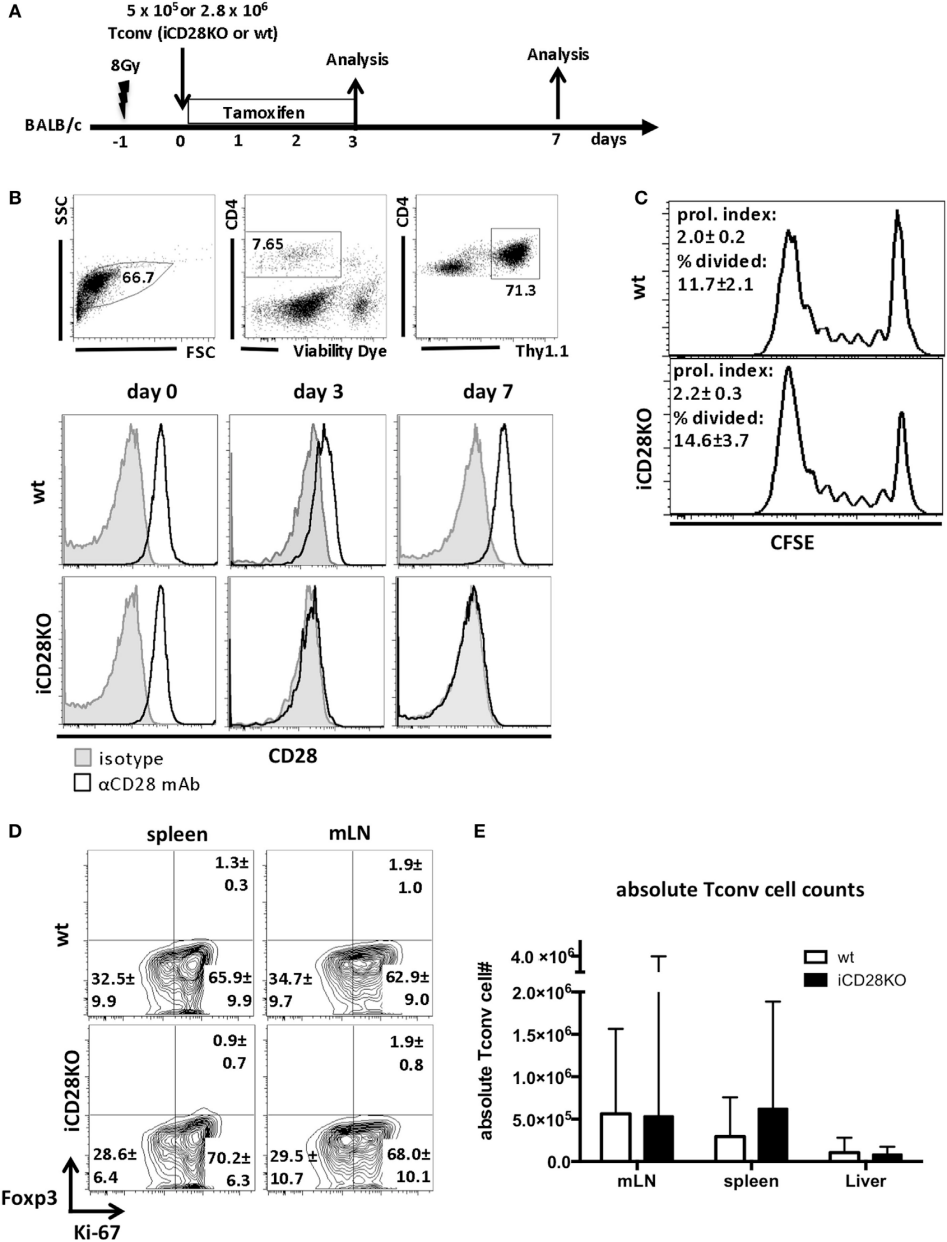
C57BL/6 donor Tconv proliferation and accumulation in BALB/c hosts during acute graft-versus-host disease is not impaired after induced CD28 deletion. **(A)** Lethally irradiated BALB/c mice were transplanted with 10^7^ C57BL/6 T cell-depleted bone marrow cells alone or together with 5 × 10^5^ unlabeled or 2.8 × 10^6^ CFSE labeled (day 3 data) Tconv from inducible CD28 knockout (iCD28KO) mice or wt littermates. CD28 deletion on iCD28KO donor Tconv was induced by tamoxifen treatment of the recipient mice from day 0 to day 3 after transplantation. **(B)** Donor Tconv were identified in the spleen of recipient mice by expression of Thy1.1 and stained for CD28 expression before transplantation (day 0) and on day 3 and day 7 after transplantation. Black histograms: specific staining. Gray histograms: istoype control staining. **(C)** CFSE dilution among splenic donor Tconv was analyzed on day 3 after transplantation. Proliferation index and percentage of divided cells are shown as mean ± SD of three independent experiments (*n* = 4 mice/group) and were tested with two-tailed Mann–Whitney test: *p* > 0.05. **(D)** Expression of Ki-67 and Foxp3 by donor iCD28KO or wt Tconv from spleen and mesenteric lymph node (mLN) of recipient mice on day 7 after transplantation. **(E)** Absolute numbers of donor Tconv per organ. Data of three independent experiments with a total of 8 mice per group are shown as mean percentages ± SD **(D)** or medians and range **(E)**. White columns: wt Tconv; black columns: iCD28KO Tconv.

We used iCD28KO mice (16, 36) as T cell donors in a fully mismatched mouse model of hematopoietic stem cell transplantation: lethally irradiated BALB/c recipient mice were reconstituted with TCD-BM from wild-type (wt) C57BL/6 mice and CD4^+^ CD25^−^ conventional T cells (Tconv) from C57BL/6 iCD28KO mice or their wt littermates. CD28 deletion on donor T cells was induced by treatment of recipient mice with 1.25 mg tamoxifen per day in a watery solution for four consecutive days, starting with the day of transplantation (Figure 1A). We chose this treatment schedule because it reflects potential clinical applications of CD28-blocking agents that would most likely also be administered post transplantation (37). On day 3 and day 7 after transplantation, Thy1.1^+^ donor Tconv recovered from the secondary lymphoid organs of the recipient mice were analyzed. Tamoxifen treatment of the recipient mice resulted in reduced CD28 expression on CD4^+^ donor T cells from day 1 after transplantation onward (data not shown), resulting in almost full ablation by day 3 after transplantation and complete CD28 deletion by day 7 after transplantation (Figure 1B).

We next assessed the proliferation of wt and iCD28KO Tconv in the allogeneic host. CFSE dilution experiments (Figure 1C) and high expression of the proliferation marker Ki-67 by donor Tconv (Figure 1D) revealed that both, wt and iCD28KO Tconv, proliferated equally in the allogeneic host after transplantation. Moreover, the transferred Tconv remained negative for Foxp3 showing that no Treg were induced (Figure 1D). CD28 deletion also did not affect the accumulation of donor Tconv in the spleens, mLNs, and livers of the hosts (Figure 1E). Moreover, we could not observe changes in the phosphorylation of Akt (pAkt), the Glut1, and the amino acid transporter 1 (LAT1, CD98) in the donor Tconv after CD28 deletion, indicating that Akt pathway activation and metabolic phenotype of the donor Tconv was fully compensated after genetic ablation of CD28 (Figures S1D–F in Supplementary Material, wt Tconv and iCD28KO Tconv without co-transfer of Treg). Taken together, our data demonstrate that treatment of recipient mice with tamoxifen starting on the day of transplantation led to inducible CD28 deletion on donor Tconv early after transplantation and that this did not impair proliferation and expansion of iCD28KO donor Tconv.

### CD28 Deletion on Donor T Cells before Transplantation Also Does Not Affect Accumulation of Allogeneic T Cells or the Phenotype of Donor Treg in Recipient Mice

**Figure 2:**
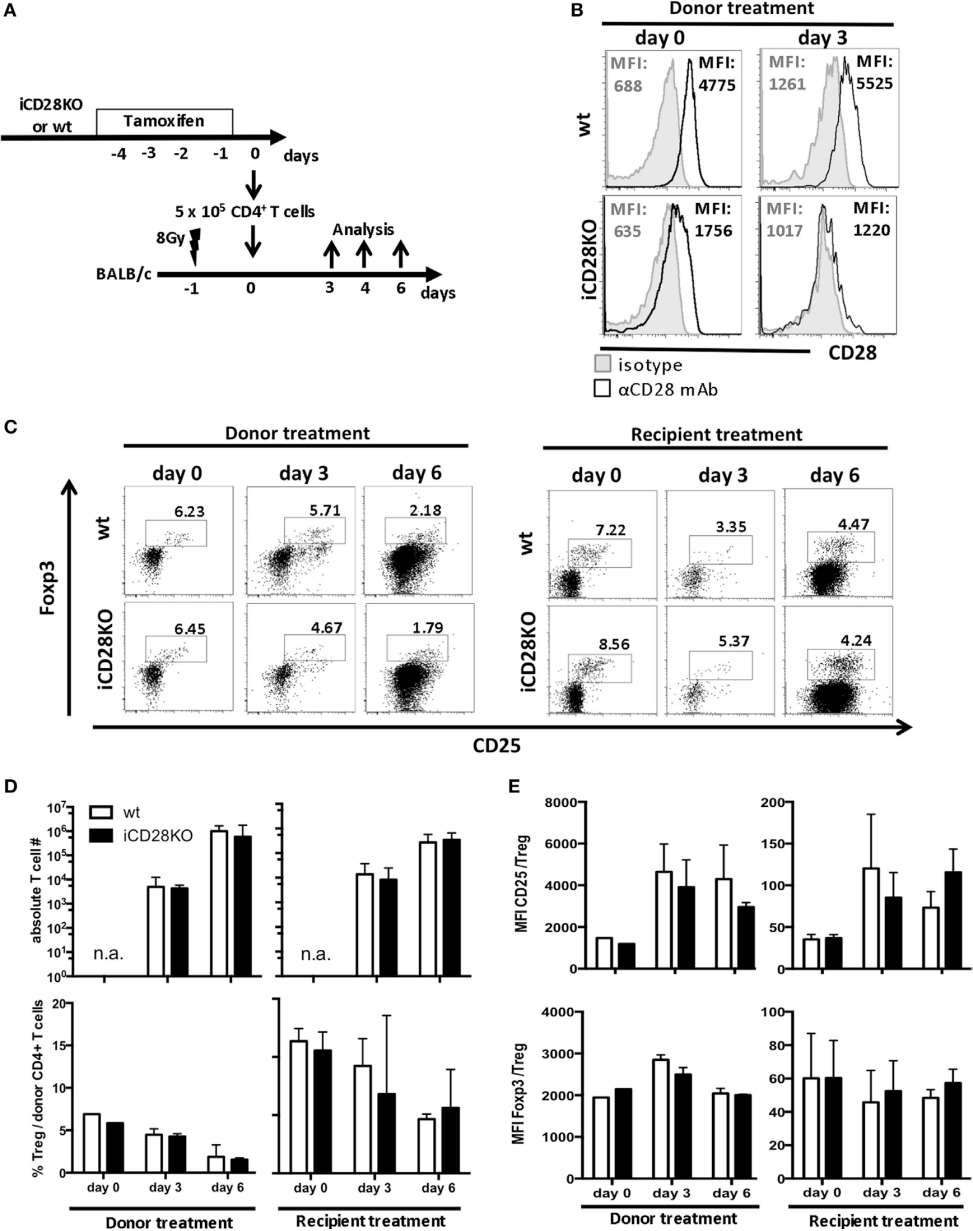
CD28 deletion on donor T cells before transplantation also does not affect accumulation of allogeneic T cells or phenotype of donor regulatory T cells (Treg) in recipient mice. **(A)** Transfer of 10^7^ C57BL/6 T cell-depleted bone marrow cells together with either 5 × 10^5^ wt or inducible CD28 knockout (iCD28KO) total CD4^+^ T cells into lethally irradiated BALB/c recipient mice. CD28 expression on donor T cells was deleted by tamoxifen treatment of the recipient mice after transplantation (see Figure 1A) or treatment of the donor mice from day −4 to day −1 before transplantation. **(B)** T cells from tamoxifen-treated donor mice were stained for CD28 expression before transfer (day 0) or 3 days after transplantation in the spleens of recipient mice [median fluorescence intensity (MFI)]. Black histograms: specific staining. Gray histograms: istoype control staining. **(C–E)** Tregs were identified by expression of CD25 and Foxp3 among freshly prepared donor T cells (day 0, before transplantation) or among splenic donor T cells (day 3 or 6 after transplantation). **(C)** Representative dot plots showing donor CD4^+^ T cells and Treg gating **(D)** Absolute donor T cell recovery from splenocytes of recipient mice and percentage of Treg among splenic donor T cells as shown in (C) (median + range). **(E)** MFI of CD25 and Foxp3 expressed by donor Treg after tamoxifen treatment of the donor or the recipient mice (mean + SD). **(D,E)** *n* = 3 recipient mice/group; white columns: wt T cells; black columns: iCD28KO T cells.

As full CD28 deletion in our model did not occur before day 3 post-transplantation, we wanted to know whether the initial presence of CD28 right after transplantation contributes to priming and activation of the donor T cells. We, therefore, compared two tamoxifen treatment schedules: tamoxifen was given either to the recipient mice directly after transplantation, as described before (Figure 1A), or to the donor mice, starting 4 days before transplantation, such that the transferred T cells had already lost 70.2% of their CD28 expression by the time of transplantation (Figures 2A,B). The residual CD28 surface expression on day 0 was not due to insufficient genetic ablation, as 3 days after transplantation, donor T cells were completely negative for CD28 by FACS staining (94.1% reduction in CD28 expression; Figure 2B). Accumulation of total CD4^+^ donor T cells in the host and the percentage of Treg among the donor T cells were independent of the treatment schedule and similar in recipients of iCD28KO and wt T cells (Figures 2C,D). However, we noticed that during the course of aGvHD development, the percentage of Treg among donor CD4^+^ T cells decreased in both, CD28-depleted and wt T cells (Figure 2D) as has been observed previously for wt T cells (22, 38). Further analysis of donor Treg revealed that during aGvHD induction expression of CD25 increased while expression of Foxp3 remained high on iCD28KO and wt Treg, independent of when CD28 deletion had been induced (Figure 2E).

Overall, there was no difference between the two treatment schedules with regard to priming or accumulation of donor T cells in the host. Moreover, CD28 deletion on donor iCD28KO T cells before or after transplantation had no effect on Treg recovery and phenotype.

### CD28 Deficiency of Donor Tconv Reduces Early Clinical Signs of Disease

**Figure 3:**
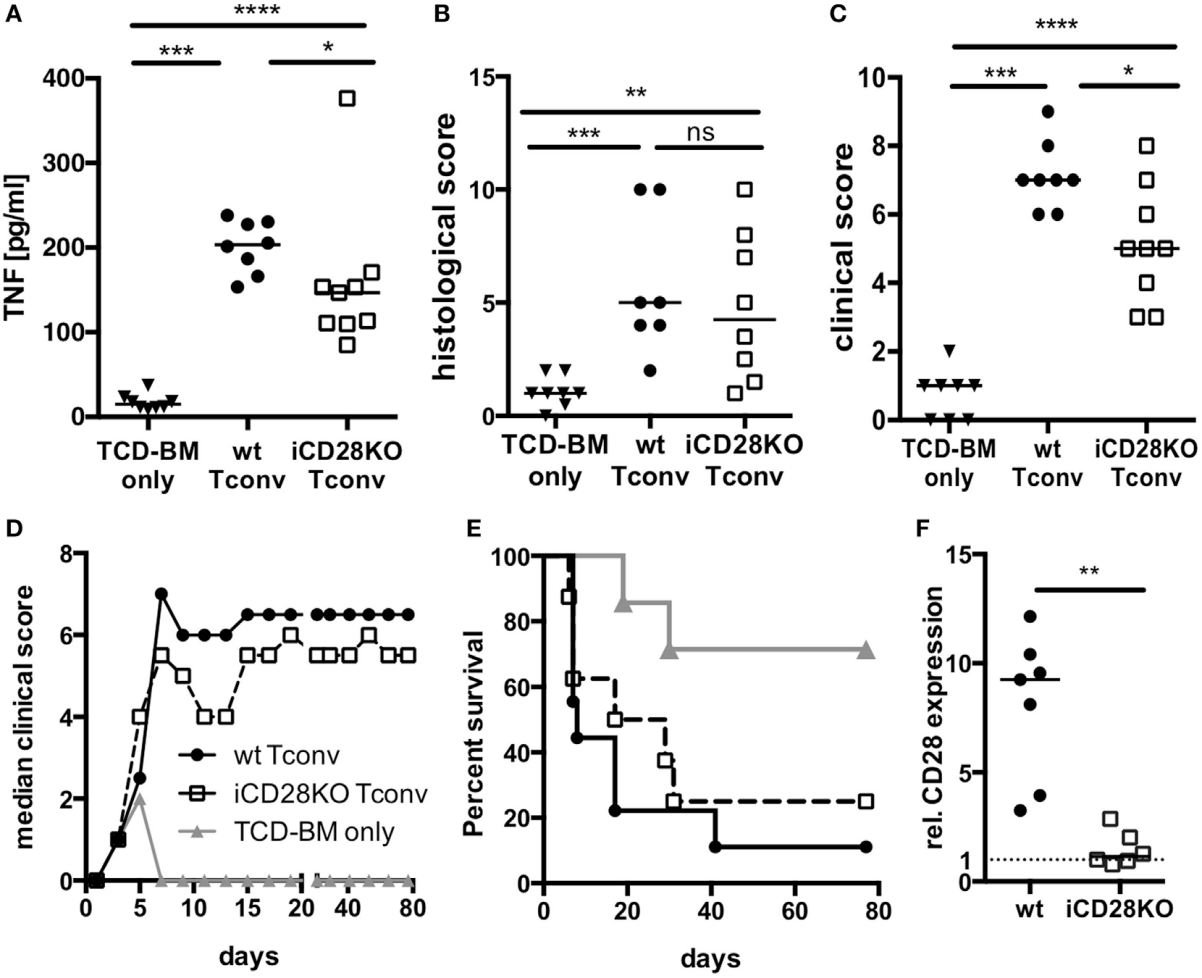
CD28 deficiency of donor Tconv reduces early clinical signs of acute graft-versus-host disease (aGvHD), but does not significantly mediate long-term protection from aGvHD-related mortality. Lethally irradiated recipients were reconstituted with 10^7^ C57BL/6 T cell-depleted bone marrow (TCD-BM) cells alone or together with 5 × 10^5^ inducible CD28 knockout (iCD28KO) or wt Tconv and treated with tamoxifen beginning on the day of transplantation. **(A)** Concentrations of TNF in the serum, **(B)** cumulative histological score of small and large bowel, and **(C)** clinical scores of recipient mice on day 7 after transplantation. Data were pooled from three independent experiments (*n* = 7–9 mice/group); two-tailed, unpaired Mann–Whitney test. **(D)** Median clinical score **(E)** survival of recipient mice and **(F)** post-mortem analysis of CD28 expression on splenic donor Tconv [rel. CD28 expression: ratio of median fluorescence intensity (MFI) of specific CD28 staining/MFI isotype control staining]. Mice that had to be killed prematurely for humane reasons are contained in the summary graph (D) until day 80 with the final clinical score assessed. Data of two independent experiments were pooled (*n* = 8 mice/group). **(A–F)** Triangles: TCD-BM only; circles: wt Tconv; squares: iCD28KO Tconv.

Since CD28-deficient donor Tconv were equally able to proliferate and expand in allogeneic recipient mice when compared to wt Tconv, we wanted to investigate if they are also similarly capable to induce inflammation and lethal aGvHD. TNF is a pro-inflammatory cytokine produced primarily by donor CD4^+^ T cells and a key player in the pathophysiology of aGvHD (39, 40). When we transferred CD4^+^ Tconv into preconditioned allogeneic recipient mice, followed by CD28 deletion (Figure 1A), TNF serum concentrations (Figure 3A) but not histopathological damage of small and large bowel (Figure 3B) were decreased in mice receiving iCD28KO Tconv as compared to mice receiving wt Tconv. Furthermore, iCD28KO Tconv recipients showed less signs of aGvHD like weight loss or diarrhea, reflected by a reduced clinical score on day 7, compared to wt Tconv recipients (Figure 3C). Since CD28 deletion on Tconv resulted in less signs of inflammation in the acute phase of the disease, we further wanted to analyze the effect of CD28 deletion on donor Tconv on recipient survival in a long-term experiment. Both, recipients of wt and iCD28KO Tconv, developed aGvHD within the first week after transplantation and finally had to be euthanized (Figures 3D,E). In recipients of iCD28KO Tconv, rapid outgrowth of ‘CD28 non-deleters’ might have caused disease, which we could, however, rule out by postmortem analysis of CD28 expression in the recipient mice (Figure 3F). Therefore, CD28-deficient Tconv were, indeed, capable of mediating lethal aGvHD. There was, however, a trend toward delayed aGvHD lethality upon CD28 deletion as the median survival was increased from 8 (wt Tconv recipients) to 23 (iCD28KO Tconv recipients) days after transplantation (Figure 3E). Taken together, our observations demonstrate that in this strongly inflammatory H-2^b^ into H-2^d^ allo-HSCT model, both, CD28-sufficient and -deficient CD4^+^ Tconv can induce lethal aGvHD. However, in the first days after transplantation, CD28 deletion on Tconv induced less inflammation in the allogeneic host when compared to wt Tconv. These observations are in line with previously published data showing that CD28 deficiency of donor T cells only has a limited effect in highly inflammatory models of aGvHD (7, 9).

### Treg Do Not Require CD28 to Suppress aGvHD during the First Week after Allo-HSCT

**Figure 4:**
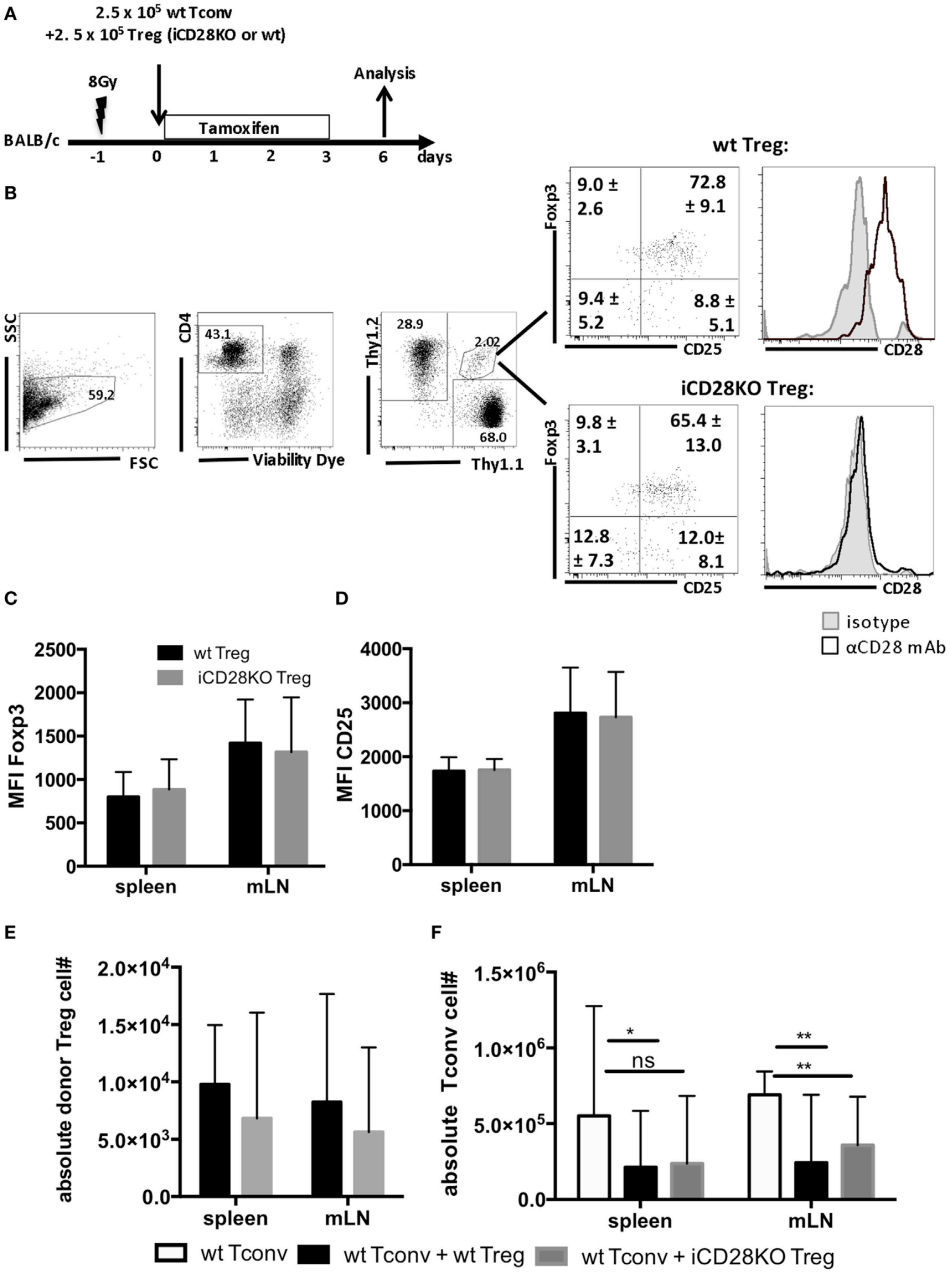
Regulatory T cells (Treg) do not require CD28 to suppress expansion of allogeneic Tconv *in vivo*. **(A)** Lethally irradiated BALB/c mice were reconstituted with T cell-depleted bone marrow cells alone or together with 2.5 × 10^5^ Thy1.1^+^ Tconv and 2.5 × 10^5^ Thy1.1^+^/Thy1.2^+^ Treg from inducible CD28 knockout (iCD28KO) donors or wt littermates and analyzed on day 6 after transplantation. **(B)** Gating strategy for identification of donor Treg in mesenteric lymph nodes (mLNs) of recipient mice and expression of Foxp3, CD25, and CD28 by donor Treg. Numbers indicate mean percentages ± SD for each quadrant. Black histograms: specific staining. Gray histograms: istoype control staining. Median fluorescence intensity of **(C)** Foxp3 and **(D)** CD25 of transferred Treg recovered from spleens and mLNs (mean + SD). **(E)** Absolute number of donor Treg and **(F)** donor Tconv per organ (median + range, one-tailed, unpaired Mann–Whitney test). **(C–F)** Data were pooled from three independent experiments, *n* = 7 mice/group. **(C–F)** white columns: wt Tconv; black columns: wt Tconv + wt Treg; gray columns: wt Tconv + iCD28KO Treg.

**Figure 5:**
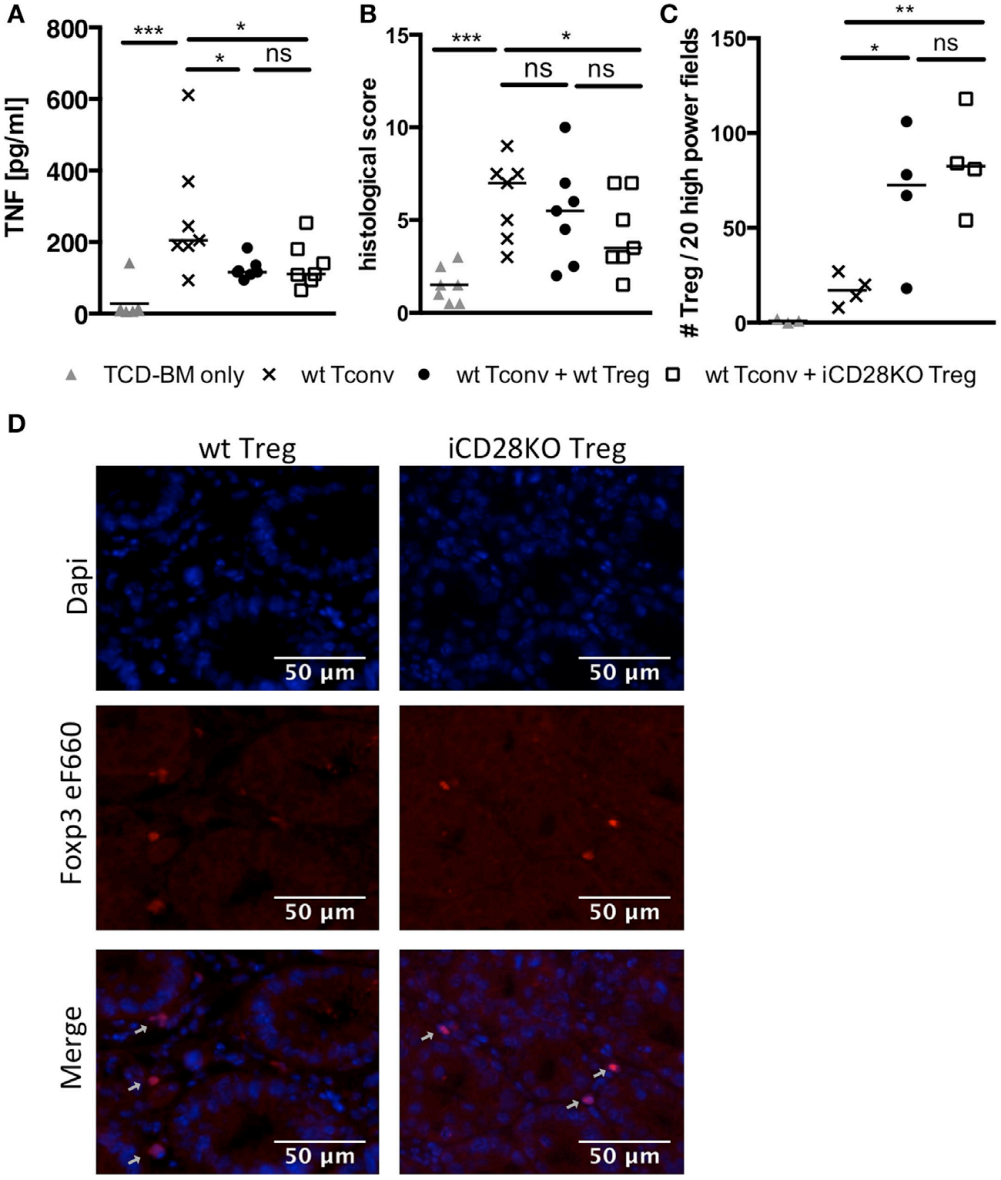
CD28-depleted regulatory T cells (Treg) can migrate to the gut and prevent tissue damage. T cell-depleted bone marrow (TCD-BM) cells were either transferred alone into BALB/c mice after lethal irradiation or together with 2.5 × 10^5^ Tconv and with or without 2.5 × 10^5^ Treg from inducible CD28 knockout (iCD28KO) donors or wt littermates. CD28 deletion was induced by treating recipient mice with tamoxifen from day 0 to day 3 after transplantation. On day 6 after transplantation **(A)** serum concentrations of TNF and **(B)** cumulative histological score of small and large bowel of recipient mice were assessed. Data were pooled from three independent experiments (*n* = 7 mice/group). **(C,D)** Paraffin sections of small and large bowel were immunohistochemically stained with Foxp3 eF660 antibody and Dapi. **(C)** Number of Foxp3 positive Treg as counted in 10 high power fields (200× magnification) of each small and large bowel. **(D)** Representative images of small bowel. Data were pooled from two independent experiments (*n* = 4 mice/group). *p* values refer to an unpaired Mann–Whitney test (comparisons between Treg recipients: two-tailed; all other comparisons: one-tailed). **(A–C)** Triangles: TCD-BM only; crosses: wt Tconv; circles: wt Tconv + wt Treg; squares: wt Tconv + iCD28KO Treg.

Donor Treg have been shown to protect mice from aGvHD (18–21), are correlated with a good prognosis in humans (41) and have, thus, started to be therapeutically utilized in patients (42). We tested the requirement for CD28 co-stimulation on Tregs for their capacity to suppress aGvHD development. For this, we co-transplanted wt Tconv and Treg from wt or iCD28KO mice at a 1:1 ratio and depleted CD28 expression on iCD28KO Treg by tamoxifen treatment of the lethally irradiated BALB/c recipient mice (Figure 4A). Similar to the deletion of CD28 on all donor CD4^+^ T cells (Figure 2E), selective knockout of CD28 on Treg only led to equally high levels of Foxp3 and CD25 expression in wt and iCD28KO Treg on day 6 after transplantation (Figures 4B–D). Furthermore, the recovery of Treg from secondary lymphoid organs on day 6 after transplantation was similar for CD28-sufficient and -deficient cells (Figure 4E). Analysis of absolute numbers of donor Tconv in the secondary lymphoid organs revealed that CD28-depleted and wt Treg were equally able to suppress Tconv accumulation in the spleen and mLNs of recipient mice (Figure 4F). Moreover, both, CD28-deficient and -sufficient Treg were able to reduce TNF concentrations (Figure 5A) and tissue damage in the gut (Figure 5B) when compared to Tconv only recipients. We also found iCD28KO and wt Treg in similar numbers in small and large bowel when analyzed by fluorescence microscopy (Figures 5C,D). This indicates that CD28 deficiency on Treg does not impair their capacity to migrate to the gut and locally prevent tissue damage (Figure 5B). Taken together, our data show that, right after allo-HSCT, CD28 deletion on donor Treg does neither impair activation of these cells in the allogeneic host nor their suppressive activity toward Tconv. Conversely, wt Treg suppressed aGvHD after transplantation of iCD28KO Tconv more strongly than after transplantation of wt Tconv (Figure S1I in Supplementary Material). The same was true at the cellular level analyzing pAkt, CD98 (LAT1) and Glut1 expression by Tconv in the presence and the absence of Treg (Figures S1D–F in Supplementary Material). However, reduced TNF serum concentrations (Figure 3; Figure S1H in Supplementary Material) and histopathological changes (Figure S1G in Supplementary Material) suggest differences in the quality, i.e., the precise pathomechanism, of wt and iCD28KO Tconv-induced aGvHD precluding a truly quantitative comparison of wt and iCD28KO Tconv regarding their susceptibility toward Treg-mediated suppression.

### CD28-Depleted Treg Fail to Mediate Long-term Protection of Recipient Mice from aGvHD

**Figure 6:**
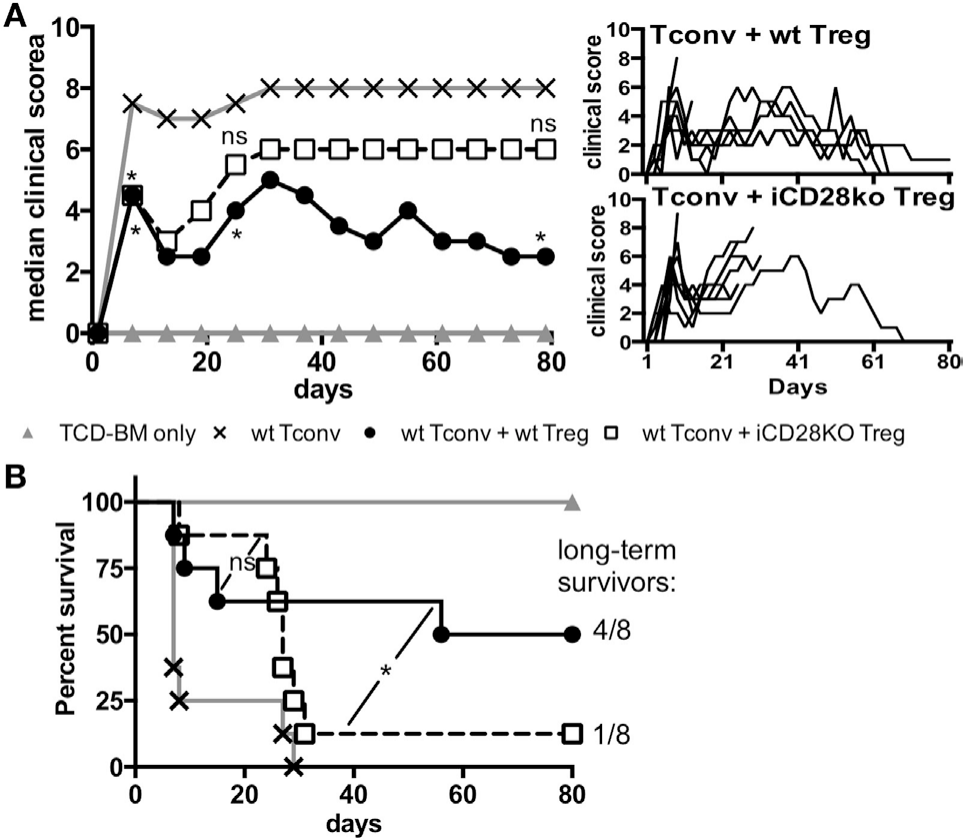
CD28-deficient donor regulatory T cells (Treg) fail to mediate long-term protection from aGvHD. Lethally irradiated recipients received either T cell-depleted bone marrow (TCD-BM) alone or together with 1.25 × 10^5^ Tconv and with or without 2.5 × 10^5^ Treg from inducible CD28 knockout (iCD28KO) donors or wt littermates. **(A)** Median clinical score (including final scores of animals that had to be killed for humane reasons) of all experimental groups (left) with individual clinical scores of Treg recipients (right). *p* values refer to a one-tailed Mann–Whitney test between Tconv only and Tconv + Treg recipients on day 7, 25, and 80. **(B)** Survival of all recipient mice and ratio of long-term survivors in the Treg recipient groups. *p* values refer to a Mantel–Cox test between the two groups receiving Treg until day 24 or from day 25 until the end of the experiment. **(A,B)** *n* = 8 mice/group; pool of two independent experiments.

As suppressive activity of iCD28KO Treg was not inferior to that of wt Treg during the first, hyperacute, phase of aGvHD we wanted to know if CD28-depleted Treg could also improve clinical symptoms and survival of the recipient mice. The presence of Treg in the transplant reduced clinical signs of aGvHD beyond the first week after allo-HSCT until about day 20 independent of CD28 expression by the Treg (Figure 6A, day 7). After day 20, however, in almost all recipients of iCD28KO Treg aGvHD strongly flared so that mice had to be killed for humane reasons whereas wt Treg continued to protect recipients from aGvHD-related mortality (Figures 6A,B). Remarkably, all long-term survivors fully recovered from aGvHD and had a maximal clinical score of 1 by the end of the experiment (Figure 6A, right panel).

### Donor Treg Require CD28 Expression to Survive until Day 19 Post Allo-HSCT

**Figure 7:**
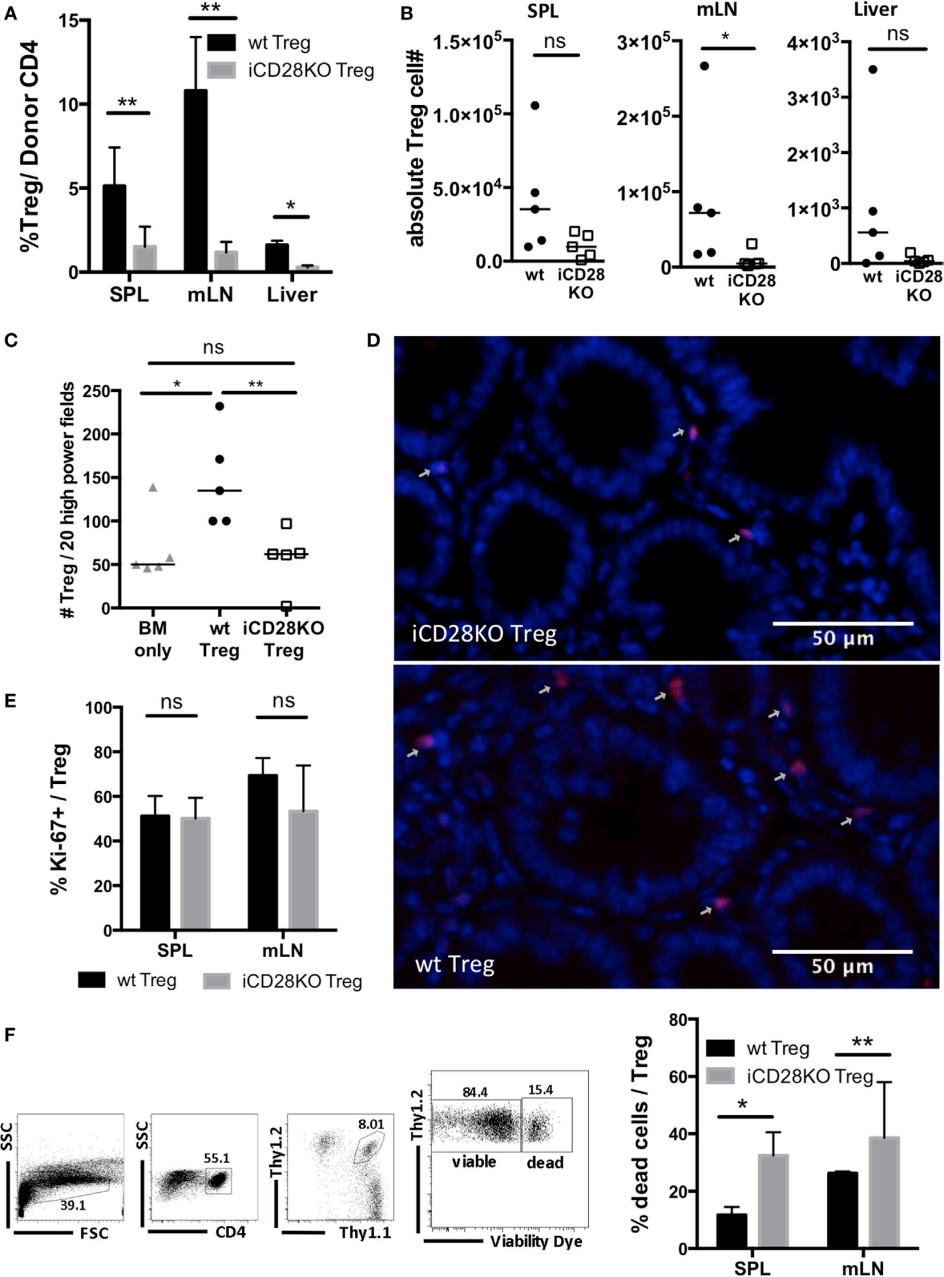
Impaired survival of CD28-deficient donor regulatory T cells (Treg) during second wave of acute graft-versus-host disease. Preconditioned BALB/c recipients received T cell-depleted bone marrow (TCD-BM) alone or together with 4 × 10^4^ Tconv and 2.5 × 10^5^ Treg from inducible CD28 knockout (iCD28KO) donors or wt littermates before tamoxifen treatment. On day 19 after transplantation, mice were sacrificed and donor Treg were identified in spleen (SPL), mesenteric lymph nodes (mLN), and liver as Thy1.1^+^Thy1.2^+^ (see Figure 4B). **(A)** Percentage of Treg among donor T cells (median + range) and **(B)** donor Treg recovery. **(C,D)** Paraffin sections of small and large bowel were stained with Foxp3 eF660 antibody and Dapi and **(C)** the number of Foxp3^+^Dapi^+^ cells assessed in 10 high power fields (200× magnification) of small and large bowel each. Triangles: TCD-BM only; circles: wt Tconv + wt Treg; squares: wt Tconv + iCD28KO Treg. **(D)** Representative overlays of Dapi and Foxp3 staining in the small bowel. **(E)** Percentage of Ki-67^+^ Treg among transferred Treg in SPL and mLN (median + range). **(F)** Gating strategy to differentiate viable and dead donor Treg (left) and percentage of dead Treg (right) in SPL and mLN (median + range). **(A,E,F)** black columns: wt Tconv + wt Treg; gray columns: wt Tconv + iCD28KO Treg. **(B,C)** circles: wt Tconv + wt Treg; squares: wt Tconv + iCD28KO Treg. **(A–F)** *n* = 4–5 mice per group. *p* values refer to a two-tailed, unpaired Mann–Whitney test.

In order to investigate why iCD28KO Treg failed to mediate long-term protection, we repeated the experiment shown in Figure 6 but sacrificed the mice on day 19, i.e., just before aGvHD had flared in the previous experiment. Donor Treg frequencies and absolute numbers of donor Treg in the spleens, mLNs, and livers of iCD28KO Treg recipients were reduced when compared to wt Treg recipients (Figures 7A,B). We also found less Foxp3^+^ Treg in the gut of iCD28KO Treg recipients compared to wt Treg recipients when we analyzed immuohistochemically stained tissue sections of small and large bowel (Figures 7C,D). Moreover, while the percentage of cycling (Ki-67^+^) cells was not reduced (Figure 7E), we detected more dead cells among donor Treg in the absence than in the presence of CD28 expression (Figure 7F). This shows that during aGvHD remission CD28 expression on donor Treg is crucial for Treg survival in the allogeneic host. In summary, our experiments demonstrate that CD28 co-stimulation on Treg is dispensable right after allo-HSCT, but crucial for Treg survival and long-term protection of recipient mice beyond about day 20 in the allo-HSCT model used in this study.

## Discussion

Using an inducible knockout model this study is the first to show that Treg require CD28 expression to survive long term in the allogeneic recipient and to mediate long-term protection from aGvHD. Defective CD28 expression and/or signaling on Treg, thus, constitutes a risk factor to develop lethal aGvHD.

In published studies, induction of aGvHD was reduced to varying degrees, depending on the exact model employed, when CD28^−/−^ mice were used as T cell donors or when CD28 signaling was blocked (7–9, 43). Accordingly, in the H-2^b^ into H-2^d^ model we used, CD28 deletion on Tconv did not abrogate aGvHD, but diminished inflammation during the first week after allo-HSCT (Figures 3A–C). With respect to the constitutively CD28-deficient mice utilized in other studies, the impact of CD28 on thymic T cell differentiation (12, 27–29), Treg maintenance in the periphery (14–17) and, related, reduced aGvHD induction by pre-activated Tconv (32, 44–47) are factors hampering the interpretation of these findings. With the iCD28KO mouse model, we could overcome the drawbacks of the constitutively CD28-deficient mice.

In contrast to what had previously been described for the blocking anti-CD28 mAb clone E18 (31) in the mouse or for the anti-human CD28 pegylated Fab fragment FR104 (48) in monkeys, inducible gene deletion of CD28 did not result in increased Treg frequencies among donor CD4^+^ T cells in our aGvHD model (Figure 2). Intrinsic agonistic activity (mAb E18) or species differences (FR104) might explain why Treg frequencies were only increased with these reagents, but not after genetic CD28 ablation.

In our experiments, loss of CD28 stimulation in Tregs did not significantly alter their phenotype or function during early aGvHD development. Neither Treg activation (Figures 2 and 4) nor migration to the gut (Figure 5) or suppression of Tconv in secondary lymphoid organs (Figure 4) was reduced when CD28-depleted Treg were compared with wt Treg. As a consequence, recipient mice of both, CD28-deficient and wt Treg, showed less aGvHD symptoms for the first 3 weeks after allo-HSCT (Figure 6). Also other disease models, analyzing T cells from constitutively CD28-deficient mice, revealed that Treg are *per se* functional *in vivo*, despite abrogated CD28 co-stimulation (17, 49, 50). With regard to Treg expansion and survival, we assume that other costimulatory signals or pro-inflammatory mediators (“signal 3”) (11) overcame the requirement for CD28 co-stimulation during the first (Figures 4 and 5) and maybe also second week after transplantation of the T cells into the recipient mice. However, when the strength of “signal 3” induced by the conditioning regimen (2, 25, 26) started to drop, CD28 co-stimulation was again necessary to maintain donor Treg numbers as is the case under steady state conditions (15–17, 51). We assume that CD28’s capacity to induce anti-apoptotic factors like Bcl-xL (52) and probably also to enhance glycolytic activity (53) maintains donor Treg from day 19 after transplantation onwards. As far as glycolysis is concerned, Treg show an overall much lower glycolytic activity than Tconv, which is necessary to maintain suppressive activity of Treg (54). Therefore, CD28-sufficient Treg which are fully functional and protect recipient animals from late-onset aGvHD are certainly still low regarding their glycolytic activity. Despite this, a drop in glycolytic activity below a certain threshold due to CD28 deficiency might negatively impact Treg survival.

For humans, there are no data on CD28 expression/signaling in Treg and the risk to develop aGvHD after allo-HSCT. However, for CD28’s inhibitory counter player CTLA-4 it has been shown that the 49G polymorphism, leading to comparatively weak B7-binding (55), is associated with enhanced T cell responses *in vitro* (55, 56) and a higher risk to develop chronic GvHD *in vivo* (57). These data are best interpreted as a lack of CTLA-4-mediated inhibition of alloreactive effector T cells causing more severe GvHD. For Treg, CTLA-4 not only is a key effector molecule for suppression (58), but also an inhibitory molecule for Treg themselves (59). Therefore, Treg expressing 49G CTLA-4 can be expected to be less inhibited by CTLA-4 and to receive enhanced CD28 co-stimulation. This might partially compensate for defective inhibition of alloreactive effector T cells expressing 49G CTLA-4. Comparing Treg phenotype and function from donors expressing 49G versus 49A (strong B7 binding) (55) would be an important next step to determine whether defective CD28 signaling in human Treg, indeed, constitutes a risk factor to develop aGvHD.

In summary, this is, to our knowledge, the first study describing a requirement for CD28 co-stimulation on Treg during aGvHD. In the absence of CD28, the donor Treg pool had largely collapsed by about 3 weeks after allo-HSCT leading to full-blown aGvHD. Transplantation of CD28-deficient Treg, thus, constitutes a clinically important new mouse model of aGvHD as it mimics similar disease courses in human patients. In fact, a substantial fraction of aGvHD patients suffers from so-called late acute GvHD that has similar symptoms as classic aGvHD but either recurs or newly develops beyond day 100 after allo-HSCT (60). Our new animal model now allows to study the responsiveness of hyperacute versus late acute, but still lethal, GvHD toward standard or experimental therapies.

## Ethics Statement

All experiments were performed in agreement with German law and approved by the Regierung von Unterfranken as the responsible authority.

## Author Contributions

AU designed research studies, conducted experiments, acquired and analyzed data, and wrote the paper. SW conducted experiments, acquired, and analyzed data. FL provided reagents. TH provided reagents and wrote the paper. TK designed research studies, analyzed data, and wrote the paper. NB designed research studies, analyzed data, and wrote the paper.

## Conflict of Interest Statement

The authors declare that the research was conducted in the absence of any commercial or financial relationships that could be construed as a potential conflict of interest.

## Supplementary Material

Figure S1 CD28-deficient donor Tconv are more susceptible to suppression by regulatory T cells (Treg) than CD28-sufficient Tconv. **(A)** Lethally irradiated BALB/c recipients were transplanted with 10^7^ T cell-depleted bone marrow (TCD-BM) cells and 2.5 × 10^5^ wt or 5 × 10^5^ inducible CD28 knockout (iCD28KO) Tconv to achieve equal acute graft-versus-host disease activity. Where indicated, magnetically sorted Treg were added to the Tconv in a 1:1 Treg:Tconv ratio. Mice were treated with tamoxifen from day 0 to day 3 and analyzed on day 7 after transplantation. **(B)** CD25 and Foxp3 expression of total CD4^+^ T cells and purified Treg before and after magnetic sort, respectively. **(C)** Absolute donor Tconv recovery from spleen and mesenteric lymph nodes are shown as median + range. Filled black columns: wt Tconv; filled gray columns: iCD28KO Tconv; open black columns: wt Tconv + Treg; open gray columns: iCD28KO Tconv + Treg. **(D)** phosphorylation of Akt (pAkt), **(E)** CD98 and **(F)** glucose transporter 1 (Glut1) expression of donor Tconv shown as median fluorescence intensity, normalized to either isotype controls **(D,E)** or staining without primary antibody **(F)**. **(G)** TNF serum concentrations, **(H)** cumulative histological scores of small and large bowel sections and **(I)** clinical scores of recipient mice. **(D–I)** filled circles: wt Tconv; open circles: wt Tconv + Treg; filled squares: iCD28KO Tconv; open squares: iCD28KO Tconv + Treg. **(B–I)** Comparisons between wt Tconv and iCD28KO Tconv recipients or Tconv and Tconv + Treg recipients were tested with two-sided or one-sided Mann–Whitney test, respectively; *n* = 5 mice/group.
