# Supplementary figures and images for: Protection of Mice from Acute Graft-versus-Host Disease Requires CD28 Co-stimulation on Donor CD4+ Foxp3+ Regulatory T Cells
Source: Front Immunol. 2017 Jun 23;8:721. doi: 10.3389/fimmu.2017.00721 (PMC5481316; doi:10.3389/fimmu.2017.00721)

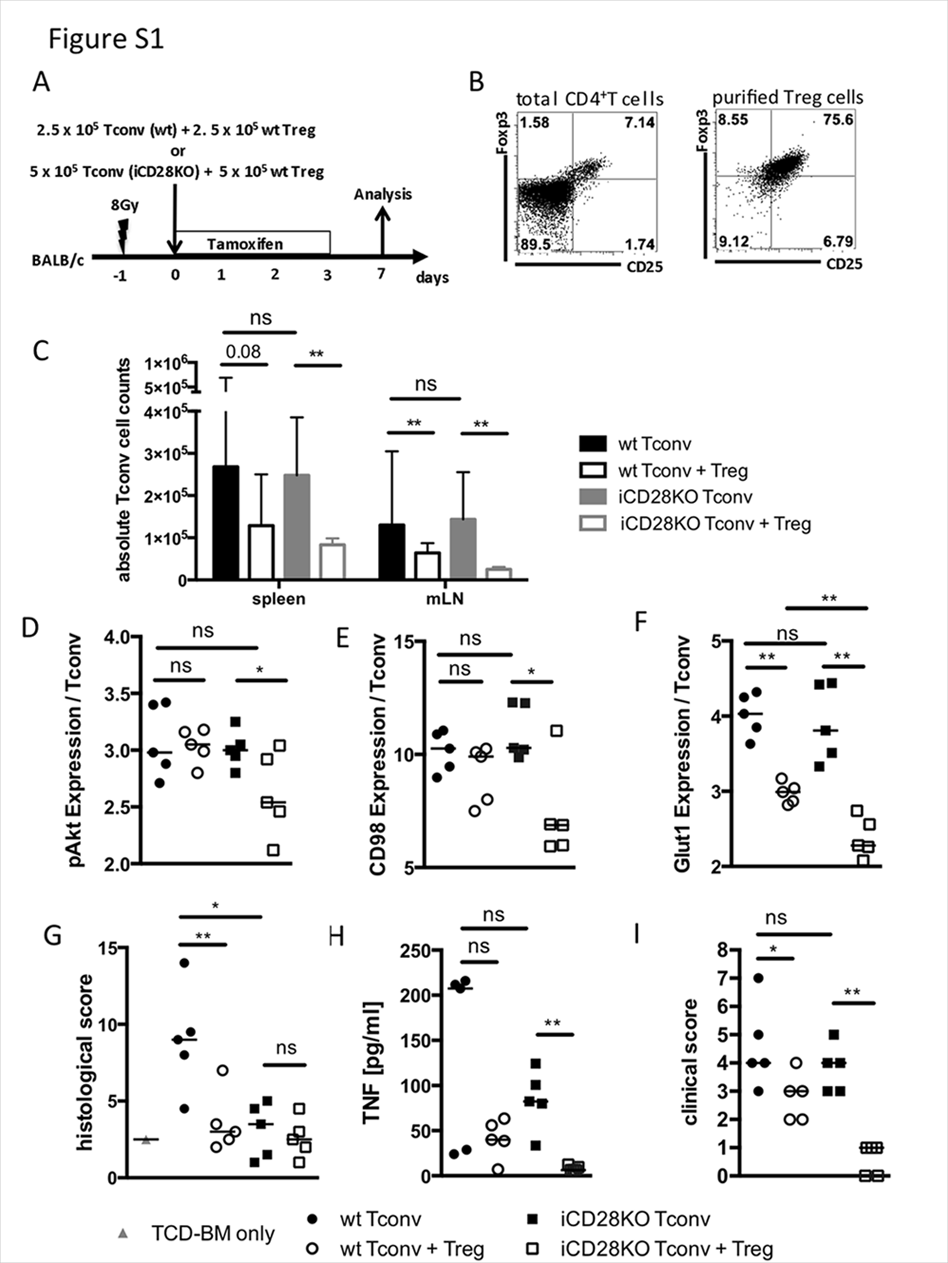

Supplement: Figure S1 — CD28-deficient donor Tconv are more susceptible to suppression by regulatory T cells (Treg) than CD28-sufficient Tconv. (A) Lethally irradiated BALB/c recipients were transplanted with 107 T cell-depleted bone marrow (TCD-BM) cells and 2.5 × 10^5^ wt or 5 × 10^5^ inducible CD28 knockout (iCD28KO) Tconv to achieve equal acute graft-versus-host disease activity. Where indicated, magnetically sorted Treg were added to the Tconv in a 1:1 Treg:Tconv ratio. Mice were treated with tamoxifen from day 0 to day 3 and analyzed on day 7 after transplantation. (B) CD25 and Foxp3 expression of total CD4+ T cells and purified Treg before and after magnetic sort, respectively. (C) Absolute donor Tconv recovery from spleen and mesenteric lymph nodes are shown as median + range. Filled black columns: wt Tconv; filled gray columns: iCD28KO Tconv; open black columns: wt Tconv + Treg; open gray columns: iCD28KO Tconv + Treg. (D) phosphorylation of Akt (pAkt), (E) CD98 and (F) glucose transporter 1 (Glut1) expression of donor Tconv shown as median fluorescence intensity, normalized to either isotype controls (D,E) or staining without primary antibody (F). (G) TNF serum concentrations, (H) cumulative histological scores of small and large bowel sections and (I) clinical scores of recipient mice. (D–I) filled circles: wt Tconv; open circles: wt Tconv + Treg; filled squares: iCD28KO Tconv; open squares: iCD28KO Tconv + Treg. (B–I) Comparisons between wt Tconv and iCD28KO Tconv recipients or Tconv and Tconv + Treg recipients were tested with two-sided or one-sided Mann–Whitney test, respectively; n = 5 mice/group. [file image_1.tif]
